# Dercum Disease: Exploratory Therapeutic Approaches in the Absence of Standardized Medical Treatment—A Single Center Case Series

**DOI:** 10.3390/life16040582

**Published:** 2026-04-01

**Authors:** Alessandro Magnatta, Alice Verdelli, Virginia Corti, Luca Sanna, Manfredi Magliulo, Valentina Ruffo di Calabria, Elisabetta Magnaterra, Elena Biancamaria Mariotti, Simone Landini, Rachel Daher, Irene Bonanni, Marta Donati, Francesca Gorini, Alessio Coi, Ilaria Di Donato, Cinzia Pupilli, Marzia Caproni

**Affiliations:** 1 Department of Health Sciences, Section of Dermatology, University of Florence, 50134 Florence, Italy; 2 Department of Health Sciences, Rare Disease Dermatological Unit, Section of Dermatology, University of Florence, P. Palagi Hospital, 50125 Florence, Italy; 3 Department of Medical and Surgical Sciences (DIMEC), University of Bologna, 40138 Bologna, Italy; 4 Azienda Usl Toscana Nord Ovest, Division of Dermatology, San Luca Hospital, 55100 Lucca, Italy; 5 Dermatology Department, University of Modena and Reggio Emilia, 41124 Modena, Italy; 6 Azienda Unità Sanitaria Locale-IRCCS di Reggio Emilia, Skin Cancer Center, 42123 Reggio Emilia, Italy; 7 Unit of Epidemiology of Rare Diseases and Congenital Anomalies, Institute of Clinical Physiology, National Research Council, 56124 Pisa, Italy; 8 Rare Neurologic Disease Center, Azienda USL Toscana Centro, San Giuseppe Hospital, 50053 Empoli, Italy; 9 SOSD Endocrinologia Azienda USL Toscana Centro, P.O. Piero Palagi Viale Michelangiolo 41, 50122 Florence, Italy

**Keywords:** Dercum disease, pain, GLP-1, tirzepatide, semaglutide, infliximab, methotrexate, treatment, weight, BMI

## Abstract

Dercum’s disease (DD) is a rare chronic disorder characterized by painful subcutaneous lipomas, mostly affecting overweight or obese middle-aged women. The etiology remains unclear, and evidence for medical treatments is limited. Surgical approaches may reduce pain but are associated with frequent relapses and are difficult to implement in extensive clinical pictures. We investigated the outcomes of multiple medical and surgical therapeutic strategies. Particularly, we explored immunomodulators (methotrexate and infliximab), used alone or combined with glucagon-like peptide-1 receptor agonists (GLP-1 RAs) such as semaglutide, as well as the dual GIP (glucose-dependent insulinotropic polypeptide)/GLP-1 RAs tirzepatide. Five patients with DD were included in this retrospective single-center case series. Baseline clinical data, medical history, and longitudinal information on Dermatology Life Quality Index (DLQI), Visual Analogue Scale (VAS) for pain, and body mass index (BMI) were collected from existing medical records and scheduled follow-up visits conducted since 2021. Clinical trajectories differed across patients and regimens. Methotrexate and infliximab coincided with variable and often transient improvements in pain and quality of life. Combination regimens including GLP-1 RAs were accompanied by weight reduction and, in selected patients, by sustained improvements in pain and DLQI. In other cases, the benefit was limited or absent. Adverse events were manageable and consistent with the known safety profiles of these drugs. In this small real-world case series, therapeutic responses in DD were highly individualized, underscoring the absence of standardized medical treatment and the need for patient-tailored strategies. The observed patterns suggest that immunomodulatory and incretin-based therapies may represent exploratory options in selected patients, especially when surgery is not feasible. However, controlled studies are needed to clarify their role.

## 1. Introduction

Dercum’s disease (DD), also known as adiposis dolorosa, is a rare, chronic, progressive disorder characterized by painful subcutaneous adipose tissue, typically presenting with multiple tender lipomas. The disease most commonly affects women aged 35–50 years, with a variable female-to-male ratio ranging from 5:1 to 30:1 [1]. DD usually occurs sporadically, though rare familial cases have been described. To date, no specific genetic mutations have been identified [2,3,4]. Its exact prevalence remains unknown.

Lipomas most frequently occur in the limbs, trunk, and back, but occasional reports of neck and head involvement have been described. DD has been classified into four types based on lesion distribution: (I) generalized diffuse (painful adipose tissue without lipomas); (II) generalized nodular (pain in the adipose tissue and in/around lipomas); (III) localized nodular (pain limited to lipomas); (IV) juxta-articular (lipomas near large joints, usually hip, knee, elbow). Mixed forms have been reported [1].

The clinical course is typically progressive. Patients often develop new lipomas over time and experience chronic pain that is frequently refractory to conventional analgesics [5,6].

After a century of debate over establishing diagnostic criteria [7], in 2012, Hansson et al. proposed a “minimal definition” of DD [1]. This definition identifies two core diagnostic elements: the coexistence of over-weight or obesity and chronically painful adipose tissue.

Neurologic and psychiatric symptoms are frequently reported. These may include sleep disturbances, impaired concentration, mild cognitive slowing, anxiety, and depression. Patients may also report constitutional symptoms such as severe asthenia, arthralgias, and myalgias.

The etiology of DD remains unclear. Multiple pathophysiological mechanisms have been proposed, including nervous system dysfunction, endocrine or metabolic disturbances, or autoimmune inflammatory processes [1,2]. Hansson et al. found altered levels of substance P, neuropeptide Y and β-endorphin in cerebrospinal fluid of affected patients [8]. Earlier hypotheses emphasized endocrine and metabolic factors [9,10], but these theories are now considered less central among current pathogenic models.

Diagnosis is primarily clinical and requires the exclusion of several mimicking conditions. These include fibromyalgia, lipedema, panniculitis, familial multiple lipomatosis, or Madelung’s disease in overweight or obese patients with multiple tender lipomas and painful adipose tissue [1,11]. Imaging techniques such as ultrasound and magnetic resonance imaging (MRI) can aid in delineating and characterizing lesions; however, no specific laboratory tests are currently available [1]. Histopathology usually shows mature adipose tissue, occasionally with inflammatory infiltrates, and connective tissue hypertrophy [12], lipogranulomatosis with multinucleated giant (MNG) cells within the adipose tissue [13,14,15], and liponecrosis [12].

DD remains difficult to manage because effective medical treatments are lacking. No standardized therapeutic protocols or long-term management guidelines currently exist.

Most therapeutic interventions are largely empirical and based on case reports or small series, while an effective long-term treatment has yet to be identified. Liposuction or surgical excision are considered the most effective treatments in reducing the pain, although recurrences are common even years later [1,5], but may be impractical in case of widespread disease. Inconsistent results have been reported with intralesional or systemic analgesics, local or intravenous lidocaine, systemic corticosteroids, transcutaneous Frequency Rhythmic Electrical Modulation System (FREMS), methotrexate, infliximab, and intralesional deoxycholic acid [1,3,5,16].

Given the hypothesized inflammatory component of DD [1], immunomodulatory therapies have been empirically attempted in selected patients. Methotrexate, a folate antagonist with anti-inflammatory and immunomodulatory properties, has been reported to improve symptoms in isolated cases [6,17]. Similarly, infliximab, a monoclonal antibody targeting tumor necrosis factor-alpha (TNF-α), has been associated with clinical improvement in a small number of case reports, sometimes in combination with methotrexate [6,17]. These observations suggest that, at least in a subset of patients, inflammatory mediators may contribute to pain and disease burden, although systematic data are lacking.

In parallel, excess adiposity may play a mechanical and metabolic role in symptom generation. Weight reduction has been associated with partial pain improvement in some patients [17,18], possibly through decreased mechanical strain and modulation of adipose tissue-related inflammatory signaling. Glucagon-like peptide-1 receptor agonists (GLP-1 RAs) and dual GIP (Glucose-dependent insulinotropic polypeptide)/GLP-1 receptor agonists are established treatments for obesity and type 2 diabetes mellitus and promote sustained weight loss. Beyond metabolic effects, these agents have been associated with the modulation of inflammatory pathways in obesity-related conditions [19,20,21,22,23,24,25,26,27,28,29]. Based on these observations, we hypothesize that these mechanisms could potentially be relevant in DD, providing a biologically plausible, but still exploratory, rationale for their use.

In this context of therapeutic uncertainty and absence of standardized management, we describe a single-center case series of patients with DD treated with different medical and surgical approaches. We focus on clinical trajectories observed with immunomodulators (methotrexate and infliximab) and incretin-based therapies. Our aim is to provide descriptive real-world observations and to explore potential therapeutic patterns in this rare and challenging condition.

## 2. Materials and Methods

This retrospective, observational, single-center case series includes all patients with DD referred to the Rare Disease Unit of the Section of Dermatology, University of Florence, between 2021 and 2025. We report real-world clinical experiences with different medical and surgical strategies in five patients with DD.

Patients were identified through the Tuscany Regional Rare Dermatological Diseases Registry. We included all consecutive eligible patients with a clinical diagnosis of DD managed at our center during the observation period. For the purpose of this report, cases were required to have: a confirmed diagnosis of DD according to Hansson’s minimal definition (chronic painful adipose tissue in the presence of overweight or obesity) [1], ultrasound evidence of multiple lipomas, receipt of at least one medical treatment at our center, and a minimum follow-up duration of 6 months.

In total, five patients were included. Two patients were excluded from this series at the time of manuscript preparation: one had not yet completed six months of follow-up during treatment, and the other, although diagnosed, had not yet received any treatment.

In accordance with routine clinical practice, the most common differential diagnoses—summarized in Table S1—were systematically excluded based on clinical findings, patient history, imaging, and histopathology when available.

All patients were examined by the same dermatology team to ensure consistency in clinical assessment. A multidisciplinary approach was adopted, involving also rheumatologists, endocrinologists, nutritionists and neurologists to ensure comprehensive patient management.

For each patient, demographic, clinical, histopathological, and biometric data, including—Dermatology Life Quality Index (DLQI), Visual Analogue Scale (VAS) for pain, and body mass index (BMI)—were routinely collected at baseline and during routine follow-up visits (typically every 3 months during the first 24 months and every 6 months thereafter, when available).

DLQI, VAS and BMI were expressed as percentage changes from baseline, summarized as individual patient trajectories, and reported in tables and figures to illustrate trends over time and across treatment regimens.

Baseline (T0) was defined as the first assessment performed at our center; the duration of symptoms prior to T0 was determined based on clinical history.

Treatments included methotrexate, infliximab, tirzepatide, semaglutide and surgery. Methotrexate was administered subcutaneously at weekly doses of 7.5–12.5 mg, adjusted according to tolerability and clinical response. Infliximab was given intravenously at 5 mg/kg at weeks 0, 2, 6 and 15, eventually followed by 120 mg subcutaneously every 2 weeks as maintenance. Tirzepatide was started at 2.5 mg once weekly and titrated up to 10 mg once weekly on the basis of clinical response and tolerability, whereas oral semaglutide was administered once daily at doses ranging from 3 to 7 mg. Surgical interventions included lipectomy (excision of individual lipomas) and liposuction. Doses and treatment sequences were individualized within the multidisciplinary team and could be reduced or discontinued in case of adverse events or poor tolerability, or switched to alternative regimens in the absence of satisfactory clinical response.

Given the small sample size and the observational nature of this single-center case series, no formal hypothesis testing was performed; data are presented using descriptive statistics only.

The study was conducted in accordance with the Declaration of Helsinki and the Italian guidance for observational drug studies (AIFA Determination No. 425/2024). Following this guidance and national regulations for non-interventional studies using anonymized clinical data, formal ethics committee approval was waived for this single-center case series. Written informed consent for the use of clinical data for research and publication was obtained from all patients. Additional consent was obtained for the publication of clinical images, where applicable.

Tirzepatide was prescribed off-label in Patient (Pt.) 5 under the treating physician’s responsibility after multidisciplinary discussion (Law 94/1998, art. 3, paragraph 2; AIFA guidance); specific written consent for off-label treatment was obtained and adverse events were monitored during follow-up.

## 3. Results

### 3.1. Clinical and Histopathological Characteristics

Five female patients with DD (age 44–65 years; median age 54 years; median age at onset 44 years; median disease duration 15 years) with baseline BMI values ranging from 24.4 to 34 kg/m^2^ were included. Two subjects were overweight (BMI > 25; <30 kg/m^2^), while one was classified as obese grade I (BMI > 30; <35 kg/m^2^). Three patients were diagnosed with form II, and two with form III.

**Figure 1:**
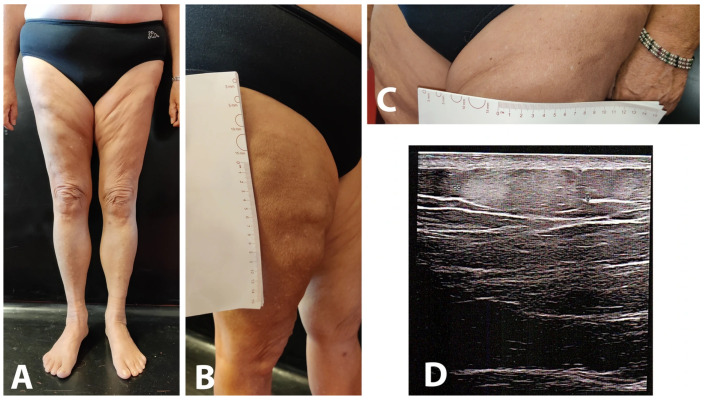
Lipomas in Pt. 2 at T30, following a 6 kg weight loss (**A**–**C**). Ultrasound imaging of a lipoma in the same patient (**D**).

Lipomas were distributed in the upper and lower limbs in all patients, predominantly in proximal regions, and were also present on the trunk in all cases. Dorsal involvement was observed in three patients, while periarticular localization was documented in one patient. Lesions ranged from numerous subcentimetric nodules (Pt. 4) to fewer discrete nodules measuring 1–6 cm in diameter. In most cases, and consistent with previous reports [30], lipomas were not clearly visible on inspection and were detectable only by palpation, particularly in patients with higher BMI (Figure 1).

Histopathological examination was available for three patients who underwent surgical procedures either before or during follow-up at our center. Pt. 1 had lipomas, whereas Pt. 3 had angiolipomas. Pt. 5 had a long history of excisions (20 lesions) performed before referral to our center, with histological diagnoses including lipomas, fibrolipomas, and a fibrolipoma arising from a nerve. Occasional additional findings included lipogranulomatosis, liponecrosis, chronic inflammation, and calcifications.

Comorbidities were heterogeneous. Pt. 1 presented with dyslipidemia and pudendal neuropathy associated with neurogenic bladder and fecal incontinence. Pt. 5 had hypothyroidism, hiatal hernia, and sigmoid diverticulosis; Pt. 4 had polycystic ovary syndrome (PCOS). Two patients had diabetes with insulin resistance (Pt. 2 and 4), and two patients (Pt. 1 and 2) had restless legs syndrome (RLS). Psychiatric comorbidities included depression in Pt. 2 and anxiety in Pt. 2 and 4.

Given the range of neurological and psychiatric symptoms reported, patients were referred to the Neurology Unit for further evaluation, including brain MRI, electroencephalogram (EEG), electromyography (EMG), and neuropsychological testing. Brain MRI demonstrated cerebral atrophy in one patient (Pt. 2) and a gliotic focus in the perinsular frontal white matter in another (Pt. 4). Slight difficulties in completing the psychological tests were also observed. Insomnia and memory impairment were each observed in three patients, while four reported concentration difficulties.

Previous treatments included non-steroidal anti-inflammatory drugs (NSAIDs), used by all patients; surgical interventions (lipoma excision or liposuction), performed in two patients; systemic cannabinoids and FREMS therapy in one patient; and amitriptyline for pain management in another.

A detailed summary of demographic characteristics, BMI, comorbidities, previous treatments, lipoma distribution, histopathological findings, and reported symptoms is provided in Table 1.

### 3.2. Management

Treatment regimens were heterogeneous across patients, differing in sequence, timing, and duration, consistent with real-world clinical practice.

Methotrexate was the most frequently administered treatment (four patients), followed by infliximab (three patients) and GLP-1 RAs (two patients treated with tirzepatide and one with semaglutide). Clinical responses varied in magnitude and duration. No reduction in lipoma size was observed in any patient; two patients (Pt. 1 and Pt. 4) experienced an increase in the number of lipomas during follow-up.

Methotrexate monotherapy was associated with heterogeneous outcomes. In two patients (Pt. 1 and Pt. 4), DLQI decreased by 81% and 70%, respectively, while VAS decreased by 57% and 43%. BMI decreased by 6.8% and 1.2% over 6 and 9 months, respectively. In contrast, Pt. 5 reported no pain reduction or quality of life (QoL) improvement, and BMI increased by 3.3%

Infliximab monotherapy showed similar variability. Pt. 1 maintained sustained low DLQI and VAS over 6 months, despite a 6.6% increase in BMI. Pt. 2 showed almost complete normalization of DLQI (−88%) and VAS (−100%) over 21 months, accompanied by a 4.3% reduction in BMI. Conversely, Pt. 3 showed only modest improvements, with maximum reductions of 17% in both DLQI and VAS and minimal BMI change (−0.4%).

Under the methotrexate plus infliximab combination regimen, DLQI, VAS and BMI decreased respectively by 91%, 100% and 1.9% in Pt. 1. A slight transient increase in DLQI was observed at month 15.

Tirzepatide plus methotrexate was associated with notable improvement in symptoms in one patient (Pt. 1), accompanied by reductions of 80% in DLQI, 75% in VAS, and 10% in BMI, sustained over 9 months (ongoing). In contrast, in another patient (Pt. 5), the same regimen was associated with minimal improvement: despite a 10% decrease in BMI, DLQI decreased by 14% and VAS remained stable. Treatment was discontinued at T7, two months after the introduction of tirzepatide (T5), due to malaise.

Similarly, during treatment with the triple combination of semaglutide, methotrexate and infliximab, DLQI decreased by 83%, VAS by 80% and BMI by 9%, persisting for 9 months (ongoing) in Pt. 2.

Four patients underwent surgical interventions (lipectomy in Pt. 1, 3 and 5; liposuction in Pt. 4). Lipectomy resulted in complete local VAS normalization (100% reduction), while liposuction achieved a 90% reduction. Lipectomy involved excision of 1–2 lipomas per session, whereas liposuction allowed treatment of a broader area. To date, recurrence in the treated region has been observed only after liposuction (Pt. 4).

**Figure 2:**
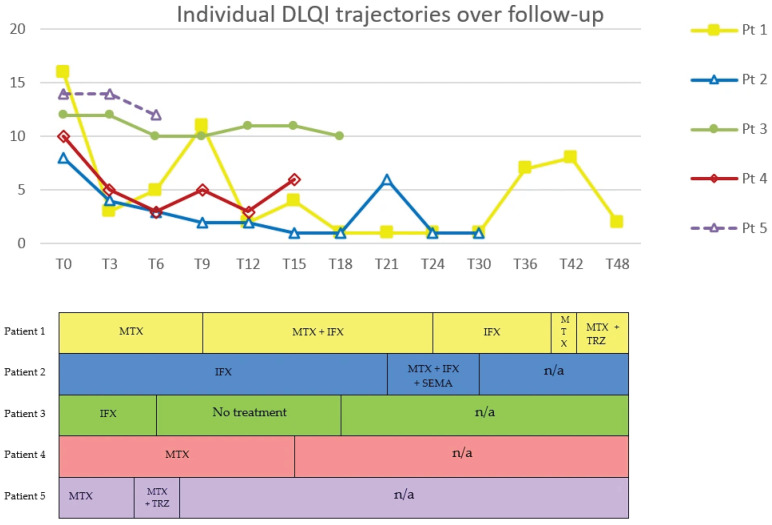
Individual DLQI trajectories over follow-up in five patients with DD. Time (months) is shown on the x-axis; T0 denotes the baseline visit (first assessment at our center). Each line represents one patient. Markers (■, △, ●, ◇) indicate follow-up visits with assessed DLQI values. Lower panel provides a schematic treatment timeline aligned to the same time points. DLQI, Dermatology Life Quality Index; MTX, methotrexate; IFX, infliximab; TRZ, tirzepatide; SEMA, semaglutide; n/a, not applicable. In Patient 1, MTX was restarted at month 39, i.e., between the T36 and T42 time points shown in the timeline. In Patient 5, TRZ was added to MTX at month 5, i.e., between the T3 and T6 time points shown in the timeline, and was suspended at T7.

**Figure 3:**
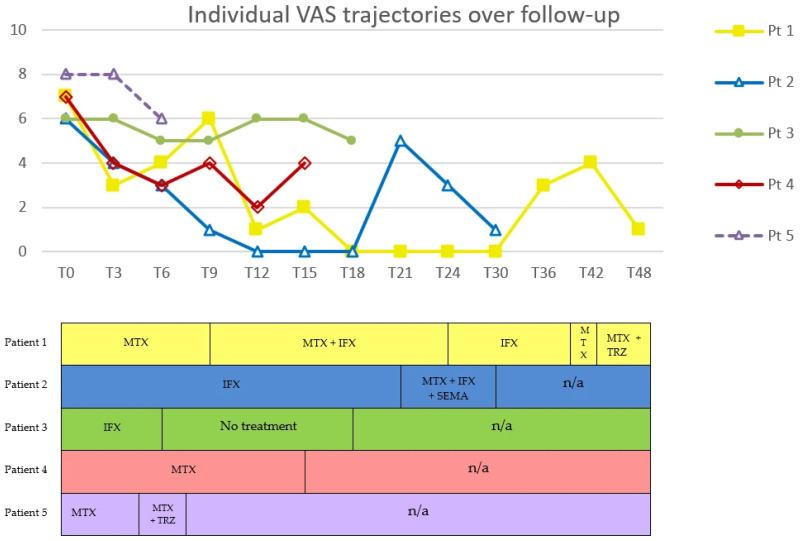
Individual VAS trajectories over follow-up in five patients with DD. Time (months) is shown on the x-axis; T0 denotes the baseline visit (first assessment at our center). Each line represents one patient. Markers (■, △, ●, ◇) indicate follow-up visits with measured VAS values. The lower panel provides a schematic treatment timeline aligned to the same time points. VAS, Visual Analogue Scale; MTX, methotrexate; IFX, infliximab; TRZ, tirzepatide; SEMA, semaglutide; n/a, not applicable. In Patient 1, MTX was restarted at month 39, i.e., between the T36 and T42 time points shown in the timeline. In Patient 5, TRZ was added to MTX at month 5, i.e., between the T3 and T6 time points shown in the timeline, and was suspended at T7.

**Figure 4:**
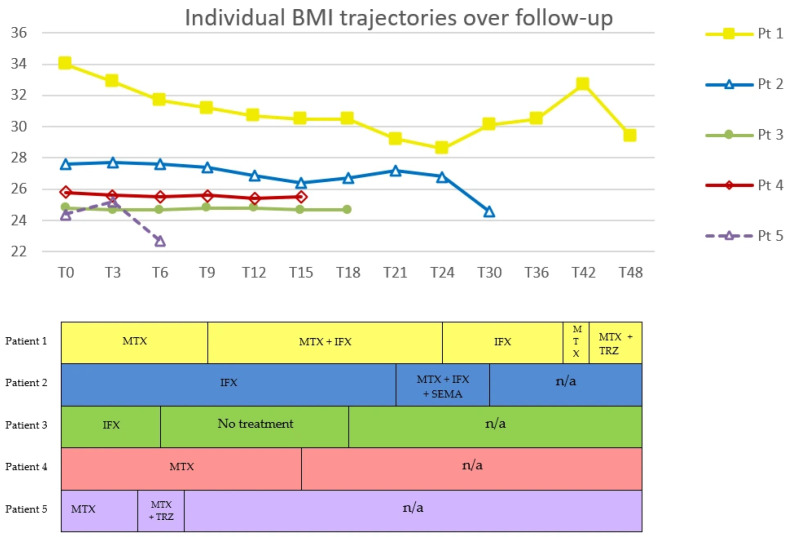
Individual BMI trajectories over follow-up in five patients with DD. BMI (kg/m^2^) is shown on the y-axis. Time (months) is shown on the x-axis; T0 denotes the baseline visit (first assessment at our center). Each line represents one patient. Markers (■, △, ●, ◇) indicate follow-up visits with measured BMI values. The lower panel provides a schematic treatment timeline aligned to the same time points. BMI, Body Mass Index; MTX, methotrexate; IFX, infliximab; TRZ, tirzepatide; SEMA, semaglutide; n/a, not applicable. In Patient 1, MTX was restarted at month 39, i.e., between the T36 and T42 time points shown in the timeline. In Patient 5, TRZ was added to MTX at month 5, i.e., between the T3 and T6 time points shown in the timeline, and was suspended at T7.

Trends in DLQI, VAS, and BMI across treatment regimens are shown in Figure 2, Figure 3 and Figure 4.

Observed clinical outcomes across medical treatment schedules, including the longest observed period with clinical benefit, are reported in Table 2. Localized post-surgery pain changes are reported in Table 3.

Drug tolerability varied across the study cohort.

Asthenia was reported in all patients treated with methotrexate (Pt. 1, Pt. 4, and Pt. 5). This adverse event led to dose reduction in Pt. 4 and treatment discontinuation in Pt. 1 at T24 due to concomitant injection-site neuralgia; symptoms subsequently resolved. Methotrexate was reintroduced in Pt. 1 at T39 without recurrence of adverse events. During methotrexate monotherapy, nausea, recurrent upper respiratory tract infections, and headache were each reported in one patient.

During treatment with GLP-1 receptor agonists, all patients experienced nausea and asthenia, with a tendency toward improvement over time. Additional adverse events included constipation and telogen effluvium in Pt. 1, which were managed through dose adjustment and supportive measures.

Infliximab was not associated with any reported adverse events in the treated patients.

Following surgical procedures, Pt. 1 and 5 experienced delayed wound healing, whereas no postoperative complications were observed in the other patients.

An overview of adverse events associated with each medical treatment and surgical intervention, stratified by patient, is provided in Table 4.

## 4. Cases’ Description

### 4.1. Patient 1

Pt. 1 had a complex medical history. Painful subcutaneous nodules first appeared in 2020, coinciding with a 20 kg weight gain. She had taken NSAIDs and amitriptyline for pain control with poor results. She presented at our center in 2021 with multiple painful lipomas (ultrasound-confirmed) on limbs and trunk, plus pain in subcutaneous tissue. We diagnosed form II DD and recorded baseline DLQI (16), VAS (7), and BMI (34 kg/m^2^).

In agreement with the rheumatologist, methotrexate (10 mg weekly) was initiated due to concomitant palindromic rheumatism, supported by the limited available evidence for DD [17]. Both cutaneous and articular symptoms improved, but subcutaneous and lipoma-associated pain progressively recurred over time.

Based on a prior case report [17], infliximab therapy was subsequently introduced at T9, using the standard psoriasis therapeutic protocol. Intravenous infliximab at a dosage of 5 mg/kg was administered at weeks 0, 2, 6, and 15, followed by subcutaneous injections (120 mg every two weeks starting at week 24). This regimen was associated with sustained reductions in pain, DLQI score, and BMI over two years. A slight transient increase in DLQI was observed at T15, possibly related to the initial absence of planned maintenance subcutaneous infliximab after intravenous infusions.

Because of asthenia and injection-site neuralgia, methotrexate was gradually tapered and discontinued at T24, with complete resolution of these adverse events.

Infliximab monotherapy subsequently showed a gradual loss of benefit and was discontinued at T36. Methotrexate at a dosage of 10 mg weekly was then restarted at T39, providing partial symptomatic benefit.

Due to ongoing weight gain despite dietary measures, tirzepatide was added (2.5 mg weekly, gradually titrated to 10 mg) at T42, with the aim of reducing mechanical strain on lipomatous tissue. This was followed by progressive pain improvement (75% reduction in VAS, 80% reduction in DLQI) and an approximately 9 kg weight loss over 6 months (BMI −10%).

### 4.2. Patient 2

Pt. 2’s history was similar to Patient 1’s. Painful subcutaneous masses first appeared 15 years before referral, but recently worsened with 20 kg weight gain. She also reported memory impairment and gait impairment. Clinical findings showed multiple discrete painful lipomas (ultrasound-confirmed) in the right popliteal region, thighs, and trunk, plus diffuse cutaneous hyperalgesia. No family history was noted. We excluded lipedema and other differentials, diagnosing mixed form II + IV DD. Her baseline DLQI, VAS, and BMI values were 8, 6, and 27.6 kg/m^2^, respectively.

The patient’s therapeutic pathway differed from Patient 1’s. Infliximab was initiated as first-line therapy and was associated with sustained reductions in DLQI (−88%), VAS (−100%) and BMI (−4.3%) over approximately two years.

At clinical worsening, methotrexate was introduced. Given her metabolic comorbidities (overweight and type 2 diabetes mellitus) and the apparent favorable response observed in Pt. 1, the endocrinologist considered adding a GLP-1 RA. Semaglutide was selected, subcutaneous methotrexate (12.5 mg once weekly) and oral semaglutide (3 mg once daily) were started concomitantly (T21).

Following gradual up-titration of semaglutide to 7 mg once daily, the patient experienced progressive pain reduction (80% reduction in VAS, 83% reduction in DLQI), together with a total weight loss of 6 kg over 9 months (BMI −10%). Given the favorable clinical response, infliximab was discontinued at T30.

### 4.3. Patient 3

Pt. 3 had a 22-year history of disease and had not received any long-term therapy, using NSAIDs only as needed. She presented at our center with multiple widespread subcutaneous painful nodules, diffuse subcutaneous pain, malaise, memory impairment, and insomnia. Following ultrasound confirmation and histologic examination (angiolipoma), we diagnosed form II DD. Baseline DLQI, VAS and BMI were respectively 12, 6 and 24.8 kg/m^2^.

Based on the favorable responses observed in the first two patients, the same intravenous infliximab regimen was initiated.

She experienced limited, transient benefit, with maximum reductions of 17% in both DLQI (2 points decrease) and VAS (1 point decrease), shortly followed by symptom exacerbation; BMI remained stable. Given the limited clinical response, the subcutaneous maintenance regimen was not pursued. The patient was subsequently lost to follow-up.

### 4.4. Patient 4

Pt. 4 presented with multiple painful, subcentimetric subcutaneous nodules involving the limbs, trunk, and back, associated with hypertrophic, congestive adipose tissue. Symptoms had begun 23 years earlier and worsened during both pregnancies and during menstruation. She had previously been diagnosed with lipedema and had undergone multiple liposuction procedures, with only transient localized benefit, followed by recurrence of nodules.

Due to the prominent symmetric lipohypertrophy of the lower limbs, the presence of the “cuff” sign (circumferential fat deposit forming a constriction at the ankle, sparing the feet), and family history of the condition, the diagnosis of lipedema was confirmed. However, given the marked pain and atypical distribution of lesions (trunk, back, and forearms), form III DD was also diagnosed. Subcutaneous methotrexate (12.5 mg weekly) was initiated, with improvements in DLQI (70% reduction), VAS (43% reduction), and BMI (1.2% decrease). At T15, the dose was gradually reduced to 7.5 mg weekly due to asthenia.

### 4.5. Patient 5

Pt. 5 presented with long-standing disease and a previous DD diagnosis from another center. Due to abdominal pain, she had undergone two exploratory laparoscopies for suspected endometriosis and abdominal hernia—both negative. Subsequent investigations eventually led to the discovery of subcutaneous lipomas. She had previously tried multiple medical and surgical treatments (see Table 1); according to the patient, the most effective—although transient—interventions were lipectomies and cannabinoids. She reported substantial weight fluctuations over the years and was overweight at diagnosis, though her BMI was normal (24.3 kg/m^2^) at our center’s first evaluation. Based on clinical findings, ultrasound and histopathologic evidence of lipomas and multiple negative lab test, we excluded differentials and confirmed the diagnosis of form III DD. Baseline DLQI and VAS were 14 and 8, respectively.

Methotrexate (10 mg weekly) was initiated first, without symptom relief over 5 months. In agreement with the patient, tirzepatide was added at 2.5 mg weekly after BMI had increased to 25.2 kg/m^2^ at T5. However, after one month and dose escalation to 5 mg weekly, she developed marked asthenia, malaise, and nausea. Due to poor tolerability and lack of clinical benefit, both tirzepatide and methotrexate were discontinued at T7, with no meaningful improvement in pain (0% VAS reduction) or QoL (14% DLQI reduction).

At T8, a new painful abdominal mass was detected on CT, raising suspicion of a desmoid tumor. Laparoscopy revealed only abdominal adhesions secondary to prior procedures, with no tumor identified. The patient reported subsequent lipectomy of two deep subcutaneous lipomas (T9), reporting immediate pain resolution (VAS −100%) in the treated area.

## 5. Discussion

### 5.1. Clinical Features

In line with previous reports [1], DD in our cohort predominantly affected middle-aged women and followed a chronic, long-standing course. The median age at symptom onset was 44 years, with a median disease duration of approximately 15 years, confirming the indolent yet persistent nature of this condition [31]. The relatively prolonged disease duration observed in our patients may reflect delayed referral to specialized centers. Diagnostic uncertainty and limited awareness of this rare disorder in routine clinical practice may also contribute.

Overweight or obesity was present in most patients (3 out of 5) at the time of the first evaluation. All patients experienced significant difficulty achieving sustained weight loss despite individualized nutritional interventions [31]. This finding is consistent with previous reports suggesting that lipomatous lesions in DD may be relatively resistant to weight reduction. Connective tissue deposition within the adipose tissue has been proposed as a potential explanation for this phenomenon [15].

Chronic pain represented the dominant symptom across the cohort. Patients typically described deep aching or tender pain localized to lipomas (clinical form III). Burning pain involving the surrounding subcutaneous adipose tissue (clinical form II) was also reported. These manifestations are consistent with previously described clinical patterns of DD and reflect the heterogeneous spectrum of pain presentations associated with this disorder [1].

In one patient (Pt. 2), superficial burning hyperesthesia of the thighs was reported and could be triggered by minimal mechanical stimulation, such as contact with bed sheets. This patient also presented with a painful lipoma in the popliteal fossa, causing knee discomfort and early limitation during walking, suggesting a mixed clinical phenotype [1]. Another patient (Pt. 4) reported diffuse itching involving the thighs. However, because pain remained localized to lipomas, she was classified as form III.

Asthenia was frequently reported and typically worsened in the evening. It limited work performance, leisure activities, and social engagement, thereby markedly affecting QoL, in agreement with previous reports [3,12,31,32]. Previous studies have suggested that fatigue in patients with DD may partly relate to obesity [1,33]; however, it is also likely influenced by chronic pain, sleep disturbances, and psychological burden. In this context, asthenia may represent a secondary consequence of the disease rather than a defining clinical feature.

Sleep disturbances were also common in our cohort, including sleep-onset insomnia, sleep-maintenance insomnia, and early morning awakening insomnia, typically associated with pain and/or anxiety, and further impaired daily functioning, as previously described [1,31,34].

Cognitive complaints, including impaired concentration and memory difficulties, were also noted in several patients, consistent with earlier findings in the literature [31,35]. Although these symptoms remain poorly characterized, they may further contribute to the overall disease burden and reduced QoL.

Brain MRI was performed in three patients and revealed nonspecific findings, including a small gliotic focus in the frontal white matter in one case (Pt. 4) and cortical atrophy in another (Pt. 2). The third examination was unremarkable (Pt. 1). The clinical significance of these findings remains uncertain, and further research is needed to clarify whether neurological alterations may be associated with DD.

Musculoskeletal symptoms were also present in our cohort. One patient (Pt. 1) presented with seronegative inflammatory polyarthritis consistent with palindromic rheumatism. Symptoms improved in parallel with adipose tissue pain during methotrexate therapy. Another patient (Pt. 2) had degenerative osteoarthritis.

Through a survey, Herbst et al. [31] found that arthralgias were frequently reported in DD, being in approximately half of the cases associated with osteoarthritis. Despite these observations, the relationship between inflammatory rheumatologic conditions and DD remains unclear.

Regarding lesion distribution, the medial proximal regions of the upper and lower limbs, the trunk, and the back were the most frequently involved anatomical areas (100%, 100%, 80% and 60% of the cohort, respectively), in agreement with previous reports [1,31]. One patient also presented with a lesion in the right popliteal fossa.

In most cases, lipomas were not readily visible during inspection and could be detected primarily through palpation, a finding consistent with earlier descriptions [3]. Interestingly, improved visibility of lipomatous lesions was observed in one patient (Pt. 2) after a 9% decrease in BMI following combination therapy with semaglutide, methotrexate and infliximab (Figure 1).

Several metabolic and psychiatric comorbidities identified in our cohort, including obesity, dyslipidemia, diabetes, insulin resistance, hypothyroidism, depression and anxiety, have previously been documented in the literature [1,31,36]. However, it remains unclear whether these conditions are directly related to DD. They may instead reflect metabolic consequences of obesity or the psychological burden associated with chronic pain. Additional comorbidities, such as PCOS and RLS, have not been previously described. In particular, two patients in our cohort presented with RLS, despite its relatively low prevalence in the general population (approximately 7%) [37]. However, it remains unclear whether this represents a true association or a coincidental finding.

### 5.2. Treatments

Given the small number of patients, as well as the heterogeneity in treatment regimens and follow-up schedules, the clinical outcomes discussed below remain exploratory and hypothesis-generating.

#### 5.2.1. Immunomodulators, Surgery and Previous Treatments

In our series, methotrexate showed variable effects. While some patients experienced meaningful reductions in pain and improvements in QoL, others derived minimal or no benefit. Similar heterogeneous responses have been reported in case-based studies, in which methotrexate demonstrated partial efficacy but inconsistent results, with many patients losing clinical response after a few months of treatment [6,17].

Consistent with previous cases [6,17], we observed favorable responses in some patients treated with infliximab. Infliximab targets TNF-α, a pro-inflammatory cytokine, suggesting a potential inflammatory component in some DD subsets. However, whether this cytokine is increased in DD remains speculative, as cytokine profiling data are lacking and warrant further investigation.

In obesity-related literature, TNF-α expression by adipocytes has been reported to be upregulated [38]. Although not directly applicable to DD, these findings support the hypothesis that adipose-driven inflammatory pathways may contribute in selected subsets. Nevertheless, in our series, targeting this pathway did not yield sustained clinical responses, as treated patients eventually worsened.

Pain recurrence in our cohort may reflect the development of neutralizing anti-infliximab antibodies, a recognized phenomenon in other inflammatory conditions [39,40]. For patient 2, antibody testing was requested, but the results are still pending.

Among surgical interventions, lipectomy provided complete local pain relief, in line with the literature [32,41,42]. Liposuction yielded a notable 90% local pain reduction (Pt. 4).

In our cohort, only liposuction was associated with local relapse. Local recurrences after lipectomy have also been reported previously [1,43], but none were observed in our series, possibly due to the relatively short postoperative follow-up.

Of previously attempted treatments, NSAIDs were the most frequently used. Although they provided transient pain relief, these agents are unsuitable for long-term management due to potential serious adverse effects. Neuromodulatory agents such as amitriptyline (Pt. 1) and therapeutic cannabis (Pt. 5) produced only partial symptomatic improvement.

#### 5.2.2. GLP-1 Receptor Agonists

To our knowledge, this is the first report describing the use of GLP-1 RAs (semaglutide and tirzepatide) in patients with DD. In our series, these agents were administered as part of combination regimens alongside methotrexate and/or infliximab. The mechanisms underlying the observed clinical effects remain unclear, and the individual contribution of each treatment component cannot be separated. Nevertheless, improvements in pain, QoL, and BMI observed in two of three patients (Pt. 1 and Pt. 2) suggest that GLP-1 RAs could represent a potentially exploitable option for DD. These findings are hypothesis-generating and should be interpreted cautiously; however, they may provide a basis for future investigation in larger cohorts.

Based on our hypothesis, Pt. 1 and Pt. 2 may have responded partly because of their clinical phenotype (overweight/obesity and form II disease). In contrast, Pt. 5, classified as form III (localized nodular disease), did not show a comparable response. In this phenotype, pain might be likely driven predominantly by focal lesion-related factors, meaning that changes in body weight may have limited clinical impact.

Clinically, GLP-1 RAs may therefore be more beneficial in forms characterized by diffuse, painful adipose tissue (forms I–II), where reducing subcutaneous fat could help relieve pressure on nerves, muscles, and tendons. Conversely, in predominantly nodular or localized forms (forms III–IV), the therapeutic effect may be less pronounced, as other weight-reduction strategies (e.g., tailored nutritional therapy) have previously failed to reduce lipoma mass in DD [15,31]. These considerations remain tentative and require confirmation in larger cohorts.

In our patients, improvements in VAS, DLQI, and BMI were observed. These findings align with the well-known metabolic effects of incretin-based therapies in obesity and type 2 diabetes. They may also reflect emerging data exploring potential anti-inflammatory effects of GLP-1 RAs in other inflammatory dermatoses and adipose tissue disorders.

Beyond metabolic effects, GLP-1–based therapies have previously been associated with reductions in systemic inflammatory markers, including C-reactive protein (CRP) and interleukin-6 (IL-6) in populations with obesity [20]. Moreover, GLP-1 RAs may improve outcomes in inflammatory dermatoses [22], including folliculitis decalvans [23], psoriasis [24,29], hidradenitis suppurativa [25], and Hailey-Hailey disease [28]. These effects may reflect both weight-related and immunomodulatory mechanisms, although current evidence remains limited to small cohorts and case-based reports.

Importantly, the inflammatory nature of DD remains incompletely defined, and our study did not include biomarker assessments, imaging endpoints, or systematic histologic analyses; thus, these mechanistic considerations should be interpreted with caution.

Additionally, an Italian case series reported encouraging results in the treatment of lipedema with the GLP-1 RA exenatide, showing reductions in body weight, pain, and subcutaneous adipose tissue thickness [26].

#### 5.2.3. Adverse Events

Most reported adverse events were consistent with the known class effects of methotrexate and GLP-1 RAs, as documented in the literature [44,45]. Methotrexate regimens were associated with dose-dependent asthenia (lasting 2–3 days, improved by dose adjustment), injection-site neuralgia, and increased susceptibility to upper respiratory tract infections, reflecting its immunosuppressive mechanism. Cephalea, although inconsistently reported [44], was also considered methotrexate-related.

No additional adverse events were observed during infliximab regimens.

Gastrointestinal manifestations are recognized effects of GLP-1 RAs. Alopecia, particularly telogen effluvium, has been increasingly recognized [46], and is likely related to rapid weight loss and subsequent nutritional imbalance, resulting in metabolic stress [47]. These events were effectively managed through dose adjustments and supportive measures.

#### 5.2.4. Final Considerations

Pain in DD is likely multifactorial. Proposed contributors include neuropathic mechanisms (e.g., local nerve irritation or mechanical compression by lipomas) and inflammatory processes in lipomatous/perilipomatous tissues, although specific triggers remain uncertain [1,48]. This heterogeneity may explain the variable responses to treatment observed across patients. Further molecular, histological, and immunohistochemical studies are needed to clarify the role of pro-inflammatory cytokines in the adipose microenvironment and whether these pathways represent actionable therapeutic targets.

In this context, it is speculative that immunomodulators such as methotrexate and infliximab may target inflammatory components, while GLP-1 RAs may influence mechanical and metabolic drivers. Weight loss has been associated with pain reduction [17,18] and with decreased TNF-α expression in obesity [49], which could reduce compression and inflammation in adipose tissue, although this has not been demonstrated specifically in DD.

A key challenge is identifying the dominant pain mechanism in each patient. This would allow for patient-tailored therapeutic strategies, which may include anti-inflammatory agents, weight-reduction interventions, intralesional lipolytics, neuromodulators, or surgery. Surgical treatment remains the primary option for single, well-defined, highly painful lesions.

## 6. Limitations

The main limitation of this study is its preliminary nature and the small, heterogeneous sample size, which largely reflects the extreme rarity of the disease.

As a retrospective case series conducted at a tertiary referral center, the study is subject to selection and reporting bias. Baseline assessments were performed at the first evaluation at our center, while pre-baseline symptoms were reconstructed from clinical history. This approach may be influenced by regression to the mean and natural disease fluctuations. However, the chronic pain trajectory of DD, characterized by exacerbations and partial remissions, reduces the likelihood that outcomes are entirely attributable to these factors.

Heterogeneous, sequential, and non–protocol-driven regimens across a small number of patients (e.g., GLP-1 RAs were never administered as monotherapy) limit the possibility of direct comparisons between therapies. The absence of placebo controls and non-standardized assessment timing further constrain interpretation and external validity. Consequently, all proposed mechanisms and treatment observations remain speculative, and the findings should be considered exploratory and hypothesis-generating rather than generalizable.

Future studies in larger cohorts should aim to standardize protocols through multicenter, multi-arm trials to enable meaningful therapeutic comparisons. These efforts could help validate our observations regarding immunomodulators and GLP-1 RAs in DD, refine treatment algorithms, and support expert consensus alongside standardized clinical guidelines.

## 7. Conclusions

DD is a rare, severely debilitating condition that is often underdiagnosed and undertreated, leading to a substantial reduction in patients’ QoL. The disease is challenging to manage, with limited evidence to guide therapy.

In this single-center series, we evaluated clinical outcomes across multiple regimens, including methotrexate and infliximab, administered as monotherapy or in combination, with semaglutide or tirzepatide added in selected patients. Clinical responses were highly variable and patient-dependent across VAS, DLQI, and BMI, ranging from minimal change to marked improvement. Adverse events were generally manageable with dose adjustments and supportive care, although one patient discontinued treatment early.

In the absence of standardized medical therapy for DD, systematically describing treatment exposures and associated response patterns can help generate speculative pathogenetic hypotheses. Methotrexate and infliximab appeared beneficial in certain patients, whereas incretin-based therapies may support long-term management through weight reduction and potential pain improvement, particularly when surgical interventions are impractical.

However, given the small sample size, concurrent therapies, and high heterogeneity in clinical presentations, treatment sequences, and follow-up durations, effects cannot be attributed to specific agents. Therefore, these findings should be interpreted strictly as descriptive and hypothesis-generating, rather than indicative of therapeutic efficacy.

Overall, reporting this case series aims to focus attention on potential new therapeutic avenues that warrant further investigation. Patient-tailored strategies targeting dominant pain mechanisms remain essential. Larger, multicenter studies are needed to validate these observations and to explore potential biomarkers, such as cytokines, for predicting treatment response.

## Figures and Tables

**Table 1:** Characteristics of the study group at the first visit. * Disease duration: interval between symptom onset and baseline visit (first assessment at our center). BMI°: assessed at baseline. BMI, Body Mass Index; N/A, not applicable; MRI, Magnetic Resonance Imaging; RLS, Restless Leg Syndrome; NSAIDs, Non-Steroidal Anti-Inflammatory Drugs, PCOS, Polycystic Ovary Syndrome; FREMS, Frequency Rhythmic Electrical Modulation System.

	Patient 1	Patient 2	Patient 3	Patient 4	Patient 5
Gender	F	F	F	F	F
Age (years)	51	65	60	44	54
Age of onset (years)	46	48	27	20	44
Disease duration * (years)	1	15	22	23	10
BMI° (kg/m^2^)	34	27.6	24.8	25.6	24.4
Clinical form	II	II + IV, mixed	II	III	III
Comorbidities	Palindromic rheumatism, pudendal neuropathy, RLS, obesity, dyslipidemia	Diabetes, depression, anxiety, RLS	N/A	Lipedema, insulin resistance, PCOS, anxiety	Hypothiroidism, hiatal hernia, diverticulosis
Previous treatments	NSAIDs, amitriptyline	NSAIDs	NSAIDs	NSAIDs, Lipectomy	NSAIDs, Cannabinoids, FREMS, lipectomy
Brain MRI	Unremarkable	Cortical atrophy	N/A	Gliotic focus in the perinsular frontal white matter	N/A
**Lipomas distribution**					
Upper limbs	+	+	+	+	+
Lower limbs	+	+	+	+	+
Trunk	+	+	−	+	+
Back	−	+	+	+	−
Periarticular	−	+ (right popliteal fossa)	−	−	−
**Histopathology**					
	Lipomas	N/A	Angiolipoma	N/A	Lipomas, fibrolipomas, liponecrosis, lipogranulomatosis, calcifications
**Symptoms complained**					
Pain	+	+	+	+	+
Insomnia	+	−	+	+	−
Impaired concentration	+	+	−	+	+
Impaired memory	+	+	+	−	−
Easy fatigability	+	+	+	+	+
Arthralgias	+	+	−	−	−

**Table 2:** Clinical outcomes across medical treatments. DLQI, VAS and BMI variations are expressed as raw (n) and percentage (%) changes. * Max benefit duration: longest observed period with clinical benefit intended as patient-reported pain reduction (assessed through VAS) and quality of life improvement (assessed through DLQI). ** No max benefit duration available for Pt.1 (Methotrexate + infliximab), as therapy was switched due to adverse events while still under clinical control. ^ Data not available because the observation period was ongoing at the last follow-up. ^§^ Discontinued due to malaise after 1 month of therapy. N/A, not applicable. DLQI, dermatology life quality index; VAS, visual analog scale; BMI, body mass index; MTX, methotrexate; IFX, infliximab; TRZ, tirzepatide; SEMA, semaglutide.

	Max DLQI Variation n (%)	Max VAS Variation n (%)	Max BMI Change n (%)	Max Benefit Duration * (Months)
**Medical treatments**				
**MTX**				
Patient 1	−13 (−81)	−4 (−57)	−2.3 (−6.8)	6
Patient 4	−7 (−70)	−3 (−43)	−0.3 (−1.2)	9
Patient 5	0 (0)	0 (0)	+0.8 (+3.3)	0
**IFX**				
Patient 1	0 (0)	0 (0)	+1.5 (+6.6)	6
Patient 2	−7 (−88)	−6 (−100)	−1.2 (−4.3)	21
Patient 3	−2 (−17)	−5 (−17)	−0.1 (−0.4)	0
**MTX + IFX**				
Patient 1	−10 (−91)	−6 (−100)	−2.6 (−1.9)	N/A **
**MTX + TRZ**				
Patient 1	−6 (−80)	−3 (−75)	−3.3 (−10)	N/A^
Patient 5	−2 (−14)	0 (0)	−2.5 (−10)	0 ^§^
**MTX + IFX + SEMA**				
Patient 2	−5 (−83)	−4 (−80)	−2.6 (−10)	N/A ^

**Table 3:** Site-specific pain variation post-surgery. * Local VAS Variation represents the change in local pain perception following surgical procedure. VAS, visual analog scale.

	Local VAS Variation * (%)	Extent of Surgery
**Surgical treatments**		
**Lipectomy**		
Patient 1	−100	2 lipomas
Patient 3	−100	1 angiolipoma
Patient 5	−100	2 lipomas
**Liposuction**		
Patient 4	−90	Wide area

**Table 4:** Adverse events were reported by each patient during medical regimens and surgical procedures over the follow-up period. MTX, methotrexate; IFX, infliximab; TRZ, tirzepatide; SEMA, semaglutide; URTIs, upper respiratory tract infections; N/A, not applicable; None, no adverse events reported. * Patient 5 reported acute abdominal pain; CT showed an abdominal mass (desmoid tumor considered; work-up incomplete due to loss to follow-up; outcome unknown).

	MTX	IFX	MTX + IFX	MTX + TRZ	MTX + IFX + SEMA	Surgery
Patient 1	Local site neuralgia, asthenia	None	Local site neuralgia, asthenia	Nausea, asthenia, constipation, telogen effluvium	N/A	Delayed wound healing
Patient 2	N/A	None	Nausea, asthenia	N/A	Nausea, asthenia	N/A
Patient 3	N/A	None	N/A	N/A	N/A	None
Patient 4	Asthenia, headache, recurrent URTIs	N/A	N/A	N/A	N/A	None
Patient 5	Asthenia	N/A	N/A	Nausea, asthenia, abdominal pain *	N/A	Delayed wound healing

## Data Availability

The original contributions presented in this study are included in the article/Supplementary Material. Further inquiries can be directed to the corresponding authors.

## Supplementary Materials

The following supporting information can be downloaded at: https://www.mdpi.com/article/10.3390/life16040582/s1, Table S1: Summary of the most relevant differential diagnoses of Dercum disease, including epidemiology, clinical features, disease course, proposed medical and/or surgical interventions, and prognosis.

## Abbreviations

The following abbreviations are used in this manuscript:

GLP-1 RAs	Glucagon-like peptide-1 receptor agonists
NSAID	Non-steroidal anti-inflammatory drug
FREMS	Frequency rhythmic electrical modulation system
DLQI	Dermatology life quality index
PCOS	Polycystic ovary syndrome
SEMA	Semaglutide
URTI	Upper respiratory tract infection
BMI	Body mass index
EEG	Electroencephalogram
EMG	Electromyography
GIP	Glucose-dependent insulinotropic polypeptide
IFX	Infliximab
MRI	Magnetic resonance imaging
MTX	Methotrexate
QoL	Quality of life
RLS	Restless leg syndrome
TNF	Tumor necrosis factor
TRZ	Tirzepatide
VAS	Visual analogue scale
DD	Dercum disease
IL	Interleukin
PT	Patient
